# Value of combining lung ultrasound score with oxygenation and functional indices in determining weaning timing for critically ill pediatric patients

**DOI:** 10.1186/s12880-025-01552-0

**Published:** 2025-01-16

**Authors:** Ximeng Hao, Hongnian Duan, Qiushuang Li, Dan Wang, Xin Yin, Zhiyan Di, Shanshan Du

**Affiliations:** 1 Department of Critical Care Medicine, Baoding Hospital, Beijing Children’s Hospital, Capital Medical University, Baoding, 071030 Hebei P.R. China; 2 Department of Ultrasound, Baoding Hospital, Beijing Children’s Hospital Affiliated to Capital Medical University, Baoding, 071030 Hebei P.R. China; 3 Baoding Children’s Severe Infectious Diseases Research Laboratory, Baoding, 071030, P.R. China

**Keywords:** Lung ultrasound score, Mechanical ventilation, Weaning, Rapid shallow breathing index, Oxygenation index

## Abstract

**Objective:**

This study aims to investigate the predictive effectiveness of bedside lung ultrasound score (LUS) in conjunction with rapid shallow breathing index (RSBI) and oxygenation index (P/F ratio) for weaning pediatric patients from mechanical ventilation.

**Methods:**

This was a retrospective study. Eighty-two critically ill pediatric patients, who were admitted to the Pediatric Intensive Care Unit (PICU) and underwent mechanical ventilation from January 2023 to April 2024, were enrolled in this study. Prior to weaning, all patients underwent bedside LUS, with concurrent measurements of their RSBI and P/F ratio. Patients were followed up for weaning outcomes and categorized into successful and failed weaning groups based on these outcomes. Differences in clinical baseline data, LUS scores, RSBI and P/F ratios between the two groups were compared. The predictive value of LUS scores, RSBI and P/F ratios for weaning outcomes was assessed using receiver operating characteristic (ROC) curves and the area under the curve (AUC).

**Results:**

Out of the 82 subjects, 73 (89.02%) successfully weaned, while 9 (10.98%) failed. No statistically significant differences were observed in age, gender, BMI, and respiratory failure-related comorbidities between the successful and failed weaning groups (*P* > 0.05). Compared to the successful weaning group, the failed weaning group exhibited longer hospital and intubation durations, higher LUS and RSBI, and lower P/F ratios, with statistically significant differences (*P* < 0.05). An LUS score ≥ 15.5 was identified as the optimal cutoff for predicting weaning failure, with superior predictive power compared to RSBI and P/F ratios. The combined use of LUS, RSBI and P/F ratios for predicting weaning outcomes yielded a larger area under the curve, indicating higher predictive efficacy.

**Conclusion:**

The LUS demonstrates a high predictive value for the weaning outcomes of pediatric patients on mechanical ventilation.

## Introduction

Mechanical ventilation is a critical intervention for saving the lives of critically ill pediatric patients, yet prolonged mechanical ventilation can lead to adverse complications such as ventilator-associated pneumonia, negatively impacting patient prognosis and increasing treatment costs [1–3]. Consequently, the clinical focus is on the early and successful weaning of pediatric patients from mechanical ventilation. In clinical practice, medical staff often rely on clinical experience to determine the timing for initiating weaning. Thus, deciding when to terminate mechanical ventilation treatment in children has emerged as a critical research question. PF ratio and RSBI are metrics traditionally used to predict weaning timing, but some studies have suggested that they have limited predictive value [4, 5]. In contrast, lung ultrasound provides more direct and real-time information on lung function. In recent years, bedside ultrasound has been widely used in predicting the weaning process because of its characteristics of non-invasive, dynamic, real-time, no need to move the patient and easy to check repeatedly. The lung ultrasound score (LUS) is a semi-quantitative assessment method that assigns scores based on the extent of loss of lung ventilation area, effectively reflecting the recovery of lung function in mechanically ventilated patients [6]. But research on it has mostly focused on adult patients. There are relatively few studies on lung ultrasound prediction weaning timing in children, but studies have shown that lung ultrasound is equally effective in assessing lung ventilation and pleural effusion in children. Therefore, it can be speculated that it also has potential value in predicting weaning timing of children [7]. This study analyzed the predictive value of lung ultrasound scores (LUS) combined with respiratory oxygenation indices for the weaning outcomes of critically ill pediatric patients.

## Materials and methods

### General data

This was a retrospective study. A cohort of 82 pediatric patients with respiratory failure undergoing mechanical ventilation in Baoding Hospital, Beijing Children’s Hospital Affiliated to Capital Medical University’s Pediatric Intensive Care Unit from January 2023 to April 2024 were selected as subjects. The study was approved by the Institutional Ethics Committee of Baoding Hospital (No. [2023]061; date: January 27th, 2023).

#### Inclusion criteria

(1) age < 18 years; (2) invasive mechanical ventilation treatment duration ≥ 48 h; (3) no use of high-dose sedatives; (4) meeting the pediatric mechanical ventilation consensus conference weaning criteria [8] (arterial oxygen tension (PaO_2_) > 60mmHg, arterial carbon dioxide tension (PaCO_2_) < 50mmHg, oxygenation index (P/F ratio) ≥ 150 mmHg, respiratory rate (f) < 30 ∼ 35 breaths/min; tidal volume (Vt) > 5mL/kg, inspired oxygen concentration (FiO_2_) < 50%, positive end-expiratory pressure (PEEP) < 5cmH_2_O, peak inspiratory pressure (PIP) < 15 cmH_2_O); and (5) normal blood pressure, stable internal environment.

#### Exclusion criteria

(1) airway stenosis, airway compression, or anatomical abnormalities; (2) large areas of skin damage or burns on the chest; (3) absence of autonomous diaphragm activity and severe muscle weakness; (4) central respiratory failure; (5) cyanotic congenital heart disease; and (6) end-stage disease or death.

### Timing of examination

To complete the weaning screening, a spontaneous breathing trial (SBT) was conducted by using low-level pressure support: 5 to 8cmH2O, FiO2 ≤ 40%, and PEEP is 0. The duration is from 30 min to 2 h. In this process, the patient’s ventilator-related indicators were continuously monitored, and the patient’s subjective feelings, such as dyspnea and anxiety, were recorded, to observe whether the patient could maintain stable autonomous breathing, and to assess its tolerance. Pulmonary ultrasound was performed 15 min after SBT began. Subjects were divided into successful and failed weaning groups based on the weaning outcomes. Reintubation within 48 h after tracheal tube removal or death of the child was considered weaning failure. The examination results were meticulously recorded, and the reasons for weaning failure were summarized and analyzed. Adjustments were made before conducting another spontaneous breathing trial and lung ultrasound examination until successful weaning was achieved.

### Collection of lung ultrasound score data

**Fig. 1:**
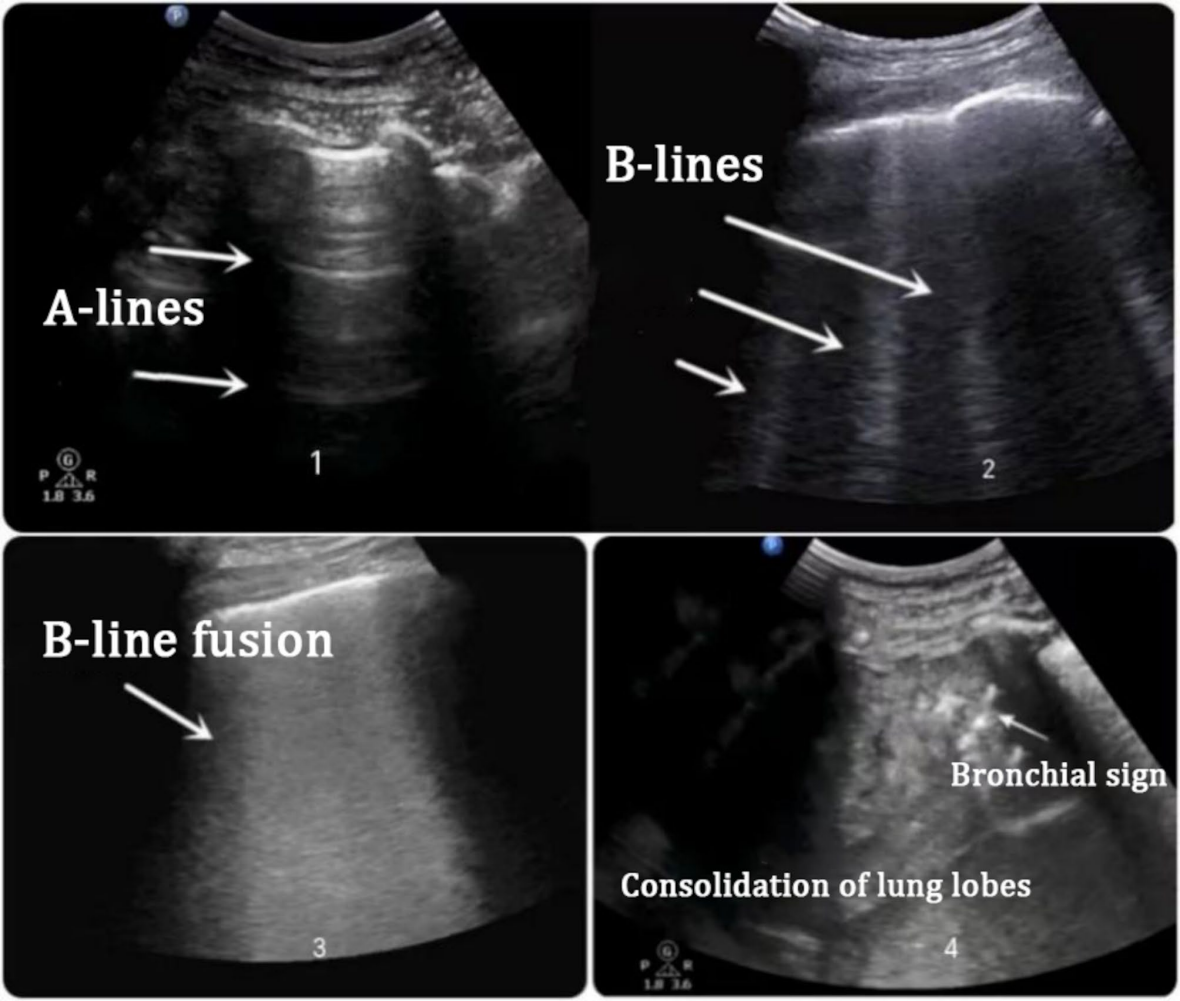
Lung Ultrasound Score (LUS) Chart. (1) A score of 0 indicates normal lung aeration (predominance of A-lines, indicative of healthy lung tissue). (2) A score of 1 signifies a moderate decrease in lung aeration (presence of 3 or more well-separated B-lines). (3) A score of 2 points to a severe reduction in lung aeration (diffuse confluent B-lines or minimal subpleural consolidation). (4) A score of 3 represents complete loss of lung aeration (liver-like appearance of lung tissue with or without air bronchograms)

Lung ultrasound was performed in all children by the same chief physician in the Color ultrasound department. A portable bedside ultrasound machine (Philips CX 50 portable Doppler ultrasound diagnostic device, L12-3 high-frequency linear array probe, frequency 3-12MHZ) was used for the examination. The lung ultrasound (atelectasis) scoring method based on body surface segmentation divided the child’s bilateral lungs into 12 regions on the body surface: anterior, middle, posterior, upper, and lower. The lung conditions of the children were assessed using the bedside ultrasound machine, and the LUS scoring criteria were based on the standards set forth in the “Technical Specification for Clinical Application of Critical Ultrasonography” [9]. LUS scoring criteria: Normal ventilation area (N): Lung sliding sign accompanied by A-lines, or < 2 separate B-lines, scored as 0; Moderate lung ventilation reduction area (B1 lines): 3 ∼ 6 B-lines present, scored as 1; Severe lung ventilation reduction area (B2 lines): Multiple ≥ 6 or densely fused B-lines, scored as 2; Lung consolidation area (C): Complete loss of ventilation, consolidation, tissue-like pattern, scored as 3. The most severe ultrasound sign in each of the 12 lung regions was assigned a score, and the sum of these scores constituted the LUS. (Fig. 1)

### Oxygenation function index data collection

Data on arterial oxygen partial pressure, inspired oxygen concentration, respiratory rate, and exhaled tidal volume were collected within 2 h prior to weaning pediatric patients from the ventilator. The oxygenation index (P/F ratio) = PaO_2_/FiO_2_. The normal value of oxygenation index is 400 ∼ 500mmHg. If PaO2 is significantly decreased, even increasing the oxygen concentration in the inhaled air will not help to further improve PaO2, when the oxygenation index is less than 300mmHg, it indicates pulmonary respiratory dysfunction. The rapid shallow breathing index (RSBI) = respiratory rate/(tidal volume/body weight in kg) were calculated. In general, RSBI < 105 is a good predictor of going weaning, meaning that the patient has a good ability to breathe on his own and is likely to successfully go weaning. If RSBI ≥ 105, it indicates that the patient may have respiratory distress or respiratory muscle fatigue, and the likelihood of successful weaning is low.

### Data collection

Clinical data collection encompassed age, gender, Body Mass Index (BMI), diagnosis of respiratory failure complications, blood gas analysis, duration of mechanical ventilation, and PICU stay duration. The weaning outcomes of the patients were subsequently followed up.

### Statistical analysis

All data were statistically analyzed using the SPSS 25.0 software (SPSS Inc., Chicago, IL, USA). Normally distributed data were represented as (‾χ ± S), and comparisons between groups were conducted using the independent samples *t*-test; non-normally distributed data were expressed as M (P25, P75), with comparisons made using the Mann-Whitney U test. Qualitative data were expressed in terms of frequency and rate, with group comparisons performed using the χ^2^ test. The final weaning outcome served as the gold standard for plotting the Receiver Operating Characteristic curve (ROC), from which the area under the curve (AUC), 95% confidence interval (CI), sensitivity, specificity were derived, and the predictive value of LUS, RSBI, P/F ratio, and their combination was determined.

## Results

### Comparison of general patient data

The ratio of male to female patients was 49/33; ages ranged from 1 to 10 years, with an average age of (6.41 ± 2.76) years. Among 82 patients, 73 (89.02%) were successfully weaned, while 9 (10.98%) failed. Weaning failure cases comprised: reconnected to mechanical ventilation after spontaneous breathing trial (*n* = 5), non-invasive ventilation within 48 h (*n* = 1), and reintubation within 48 h (*n* = 3). No statistically significant differences were observed in age, gender, BMI, and comorbidities between the successful and failed weaning groups (*P* > 0.05); however, the duration of intubation and PICU stay were significantly longer in the failed weaning group (*P* < 0.05). See Table 1 for details.

**Table 1:** Basic clinical data of patients [*n* (%)]

Clinical characteristics	Successful weaning group (*n* = 73)	Failed weaning group (*n* = 9)	Statistic	*P*
Age (years)	7.12 ± 2.23	7.01 ± 2.14	0.084	>0.05
Gender				
Male	44	5		>0.05
Female	29	4		
BMI (kg/m²)	17.22 ± 2.34	17.85 ± 3.23	0.145	>0.05
Diagnosis of respiratory failure				
Severe pneumonia	27(36.99%)	4(44.44%)		>0.05
Congenital heart disease	27(36.99%)	3(33.33%)		
Sepsis	12(16.44%)	1(11.11%)		
Hematologic malignancy	7(9.59%)	1(11.11%)		
PICU stay/d	11(3, 14)	23(14, 29)	5.244	<0.05
Duration of intubation/d	5(2, 7)	12(5, 14)	4.214	<0.05

### Comparison of lung ultrasound scores (LUS), RSBI and P/F ratio before extubation between the successful and failed weaning groups

The RSBI values and LUS were lower, while the P/F ratio was higher in the successful weaning group compared to the failed weaning group, with statistically significant differences (*P* < 0.05). See Table 2 for details.

**Table 2:** Comparison of LUS, RSBI, and P/F ratio between groups ($\overline \chi \pm S$)

Group	Number of cases	RSBI	LUS/point	*P*/F
Successful weaning group (n=)	83	4.67 ± 1.25	12.35 ± 1.02	287.45 ± 19.89
Failed weaning group (n=)	9	7.98 ± 1.24	16.92 ± 1.54	205 ± 14.45
t		4.893	8.564	6.832
*p*		<0.05	<0.05	<0.05

### Predictive analysis of LUS, RSBI and P/F ratio for weaning outcome from mechanical ventilation

ROC curve analysis was performed for LUS, RSBI and P/F ratio based on different weaning outcomes. According to the Youden index and AUC values of each assessment indicator, the predictive efficacy of LUS for weaning failure was superior to that of the RSBI and P/F ratio. The optimal threshold for predicting weaning failure was identified as LUS ≥ 15.5, which demonstrated better predictive efficacy than RSBI and P/F ratio. When LUS was combined with RSBI and P/F ratio for predicting weaning outcomes, a larger area under the curve was observed, indicating higher predictive efficacy. See Tables 3 and 4 for details.

**Table 3:** Evaluation of predictive efficacy of various parameters for weaning failure

Parameters	LUS	RSBI	*P*/F	LUS + RSBI + *P*/F
Cut-off value	≥ 15.5	≤ 216.0	>7.8	>0.1
Sensitivity/%	89.21	66.78	78.21	89.14
Specificity/%	95.91	91.24	90.78	92.45

**Table 4:** Comparison of AUC for predicting weaning outcomes among parameters

Parameters	AUC	Youden index	95%CI
LUS	0.934	0.712	0.946 ∼ 1.000
RSBI	0.756	0.543	0.883 ∼ 0.984
P/F	0.812	0.467	0.885 ∼ 0.982
LUS + RSBI + P/F	0.967	0.836	0.951 ∼ 1.000

## Discussion

Mechanical ventilatory support is a pivotal therapeutic intervention in pediatric intensive care units, safeguarding stable vital signs in children, with acute respiratory failure being the principal cause for tracheal intubation [10, 11]. The scarcity of predictive indicators for assessing the readiness of pediatric critically ill patients for ventilator weaning, coupled with inconsistent study outcomes, underscores the necessity for a standardized weaning protocol.

This study reveals that weaning outcomes are not significantly correlated with the age, body mass index, or disease type of the patients but are associated with the duration of intubation and the length of stay in the PICU. This suggests that the severity of the child’s illness is linked to the weaning outcome, highlighting that alleviation of the disease and stabilization of the child’s critical condition are prerequisites for successful weaning from mechanical ventilation. A multicenter prospective study has indicated that the risk of mortality increases with each additional day of mechanical ventilation beyond 72 h [12]. The American guidelines for weaning critically ill patients recommend assessing the basic readiness for weaning 48 h after initiating mechanical ventilation, advocating for a protocolized weaning approach based on spontaneous breathing trials (SBT) [13]. However, these findings are based on adult studies, and studies have shown that protocolized weaning does not significantly reduce the duration of intubation in critically ill children, and the rate of weaning failure is higher due to children’s weaker stress response to illness [14, 15]. The weaning failure rate in this study was 10.98%, similar to the rate previously reported in the literature [16].

In recent years, more and more studies have confirmed the diagnostic value of ultrasound in a variety of lung diseases, especially in emergency ultrasonography. Pulmonary ultrasound has great application value in the differential diagnosis of pulmonary exudative lesions, and has good sensitivity and specificity for the diagnosis of various lung diseases in newborns and children. In some cases, lung ultrasound can replace chest CT for routine diagnosis of lung disease in emergency intensive medicine, and there is an international consensus. Previous research has often focused on predicting the success of weaning using the oxygenation index (P/F ratio) and the rapid shallow breathing index (RSBI), which can help ascertain whether the basic conditions for weaning are met [17, 18]. However, no clear risk range for weaning failure has been defined. Respiratory muscle weakness can lead to severe dyspnea, and studies have shown that diaphragmatic injury caused by mechanical ventilation is closely related to weaning failure [19]. Lung ultrasound can objectively reflect the progression of lung diseases, and the bedside lung ultrasound score (LUS) is a non-invasive, real-time, semi-quantitative assessment method that assigns scores based on the extent of ventilated lung area loss [9]. A higher LUS indicates more loss of lung ventilation area and a more severe lung condition [20]. Oxygenation indicators reflect the overall gas exchange and oxygenation status of the patient, which can be influenced by ventilator settings. This study shows that the lung ultrasound score (LUS) and the rapid shallow breathing index (RSBI) were higher in the weaning failure group, while the oxygenation index (P/F ratio) was lower compared to the successful weaning group (*P* < 0.05). This indicates that the recovery of lung ventilation area or oxygenation function in the weaning failure group was inferior to that in the successful group. Some lung areas that had not regained function compensated for the deficiency in respiratory oxygenation function through low-level positive pressure ventilation, and after the removal of positive pressure support, the oxygenation function of the unrecovered lung tissue was lost, leading to weaning failure.

This study employed ROC curve analysis to compare the predictive effects of LUS, RSBI, and P/F ratio on weaning outcomes. The results showed that using LUS alone to predict weaning failure had an AUC of 0.934, a sensitivity of 89.21%, and a specificity of 95.91%, which was significantly more predictive than RSBI and P/F ratio. The results of a Spanish study found that the sensitivity and specificity of LUS in predicting extubation failure in a single factor analysis were 66% and 92%, respectively, slightly lower than the results of this study [21]. When LUS was combined with RSBI and P/F ratio for prediction, the AUC was 0.967, with a sensitivity of 89.14% and a specificity of 92.45%, indicating a larger area under the curve and higher predictive efficiency than using LUS alone. This suggests that the weaning process in children is complex, necessitating the combined application of multiple parameters for assessment, prediction, and management. Changes in lung ultrasound imaging are more sensitive than changes in oxygenation indicators, and using the lung ultrasound score (LUS) before weaning can accurately assess the recovery of lung ventilation area and the severity of lung disease without interference from positive pressure ventilation [22, 23]. This study shows that a lung ultrasound score above 15.5 points is the optimal threshold for predicting weaning failure in children. Previous studies in adult patients [24] predicted a cutoff value of LUS ≥ 17 points for weaning failure and proposed LUS < 13 as a good indicator for successful weaning. The difference in cutoff values between this study and previous studies may be related to differences in the age of the study population, but all studies found that patients with lower LUS values had a higher success rate of weaning, indicating that LUS has great potential in decision-making during the weaning process of mechanically ventilated children. Additionally, bedside lung ultrasound scoring has the advantage of being radiation-free compared to lung CT and X-rays, and it can monitor the specific location of lung lesions in real-time and dynamically, guiding clinical rehabilitation treatment in corresponding body surface areas [25].

### Limitations of this study

There are still some shortcomings in this study. This study was a retrospective descriptive study, with limited clinical data available and limited persuasive conclusions. In addition, diaphragmatic injury caused by mechanical ventilation is also closely related to weaning failure. However, this study only evaluated the value of pulmonary ultrasound in predicting weaning, which may have missed the cause of weaning failure caused by respiratory muscle problems in children.

## Conclusions

To conclude, bedside lung ultrasound scoring (LUS) can assess the lung condition of critically ill mechanically ventilated children and predict weaning outcomes. Combined with respiratory oxygenation indicators, it can provide a more comprehensive assessment and preparation for weaning in children.

## Data Availability

The datasets generated and analysed during the current study are not publicly available due to privacy but are available from the corresponding author on reasonable request.
